# Analyzing and predicting the LNM rate and prognosis of patients with intraductal papillary mucinous neoplasm of the pancreas

**DOI:** 10.1002/cam4.3632

**Published:** 2021-02-27

**Authors:** Chao‐Tao Tang, Bi‐Xia Liu, Youxiang Chen, Chunyan Zeng

**Affiliations:** ^1^ Department of Gastroenterology the First Affiliated Hospital of Nanchang University Nanchang China

**Keywords:** intraductal papillary mucinous neoplasm (IPMN), LNM, nomogram, survival, validation study

## Abstract

**Background:**

Current the surveillance and management are controversial for patients with IPMN. We aimed to develop an alternative nomogram to individualize IPMN prognosis and LNM.

**Methods:**

Based on the data from SEER database of patients diagnosed with IPMN between 2004 and 2015, a nomogram predicting the survival and LNM of IPMN based on univariate and multivariate and Lasso regression analysis was performed, internally and externally validated, and measured by C‐index, and decision curve analysis (DCA), and compared to the 7^th^ TNM stage.

**Results:**

A total of 941 patients were included. Age, T stage examined nodes, tumor size, and pathology grade were identified as an independent factor for predicting LNM. The nomogram we established to predict LNM had a high predicting value with a C‐index value of 0.735 and an AUC value of 0.753. Interestingly, including T1 stage, we found an inverse correlation was between age and LNM. In addition, nomogram for predicting CSS also performed better than TNM stage both in the internal validation group (1‐year AUC:0.753 vs. 0.693, 3‐year AUC: 0.801 vs. 0.731, 5‐year AUC: 0.803 vs. 0.733) and external validation group (1‐year AUC: 0.761 vs. 0.701, 3‐year AUC: 0.772 vs. 0.713, 5‐year AUC:0.811 vs. 0.735). DCA analysis showed the nomogram showed a greater benefit across the period of follow‐up compared to 7^th^ TNM stage.

**Conclusion:**

A nomogram based on multivariate and Lasso regression analysis showed great clinical usability compared with current criteria. Also, for LNM of IPMN, younger age patients with IPMN should be attached more importance.

## Graphical abstract

First, we build a nomogram to predict lymph node metastasis of patients with IPMN, which had a high predicting value with an C‐index value of 0.735 and an AUC of 0.753. Second, mutivariate logistic regression analysis showed age was an independent factor and heat map showed patients aged 20–39 had highest lymph node metastasis rate while patients aged >=80 had least rate. Moreover, subgroup analysis showed patients aged 20–39 years had highest LNM rate while patients aged >=80 had the lowest LNM rate in most subgroups except some values were doubted. Third, we performed Lasso regression analysis and identified the features that are highly associated with survival, at the same time, nomogram was performed based on multivariate cox regression analysis, internally and externally validated, and measured by ROC curve, C‐index, and decision curve analysis (DCA), and compared to the 7th TNM stage. DCA analysis showed no matter in the internal cohort or external cohort, the nomogram showed a greater benefit across the period of follow‐up compared to 7th TNM stage.

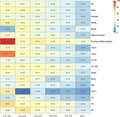

## 1 INTRODUCTION

Since 1996, intraductal papillary mucinous neoplasm (IPMN) of pancreas was recognized as an independent disease in the world.^1^ IPMN, characterized by premalignant mucin‐producing epithelial neoplasm, was divided into three categories according to tumor locations: main duct, branch duct, and mixed type.^1^ Some retrospective studies found IPMN accounted for 15%–30% cases for all cystic lesions in the pancreas and pancreatic cancers originated from IPMN accounted for 20–30%.^2^ Over the past two decades, the incidence of IPMN has augmented because the alteration of diagnosed methods such as high‐resolution diagnostic imaging including endoscopy,^3^ which ranges from 2% to 45%.^4^ Recent report showed approximately 25% resected IPMN lesion were invasive lesion demonstrated by histology.^5^ To our knowledge, due to the malignancy risk, it has to carry out surveillance for those patients with high risk to progress into tumors or to perform surgery timely. Actually, many patients become invasive during follow‐up and recurrent after surgery, suggesting it is insufficient to identify which factors are correlated with invasion and appropriate surgery.^6^, ^7^ The overall recurrence rate was different from different lesions, for example, T1 stage was with a rate of 42.9% while invasive IPMN was with a rate of 57.1%, and multivariate analysis revealed LNM was an independent risk for recurrence.^8^ In addition, as for the survival of IPMN, according to the malignance which could be identified by the pathological grade and tumor size, the overall 5‐year survival rate are 29%–92%.^9^ The existing guidelines such as International Association of Pancreatotomy (IAP) guidelines, European Study Group on Cystic Tumors of the Pancreas and American Gastroenterological Association (AGA) clinical guideline were controversial regarding to the evaluation and management of IPMN.^10^, ^11^ For instance, when and to whom surgery should be offered and when to favor surveillance was different.^12^ The frequency, duration, and modality of surveillance is also controversial, as this strategy is resource‐consuming and must be balanced against the perceived benefits and risks involved.^12^According to the European evidence‐based guidelines,^13^ it is a consensus to have correct management to prevent progression into pancreatic cancer for IPMN, moreover, it could reduce the related cost and promote survival. However, there is not an effective evaluation measures according to characteristics of lesions. Based on these issues, it is significantly meaningful to build clinical model to predict the invasion and survival of patients with IPMN.

In our study, we extracted a number of 941 patients from the Surveillance, Epidemiology, and End Results (SEER) database to investigate the lymph node metastasis (LNM) and survival of patients with IPMN. Furthermore, we performed comprehensive analysis and constructed nomogram to predict LNM rate and survival. As we all know, nomograms are widely used for assessing the prognosis of cancers because of their ability to transform a statistical predictive model into a single numerical estimate of the probability of an event, which is a user‐friendly method that guides clinical decision‐making for doctors.^14^

## 2 METHODS

### 2.1 Patients

All patients with pancreatic cancer were retrieved from the SEER database with the National Cancer Institute's SEER*Stat software (version 8.3.6). Patient was performed surgery without chemotherapy. The patients did not give informed consent because the SEER database is free for public use. According to the International Classification of Diseases in Oncology (ICD‐O‐3), tumors with codes 8050, 8260, 8450, 8453, 8471, 8480, 8481, and 8503 are identified as IPMN.^15^ In our study, patients with IPMN were included according to the following criteria: (a) patients older than 20 who were diagnosed as IPMN by positive histology from 2004 to 2015; (b) patients who have information of T stage (not including Tis), N stage and M stage; and (c) patients with detailed information, including race, pathological grade, regional nodes examined, tumor size, sex, and survival information.

### 2.2 Clinicopathological factors

The clinicopathological variables extracted from the SEER database in our study included age, race, sex, pathology grade, T stage, M stage, tumor size, N stage, regional nodes examined, and primary site. The patients were divided into six age groups: 20–39 years, 40–49 years, 50–59, 60–69 years, 70–79 years, and ≥80 years. Race was classified into three types: white, black and other. Sex included male and female. Pathology grade was categorized as well/moderately differentiated type and poorly differentiated/undifferentiated type. N stage was described as N1 (Yes) or N0 (No). M1 (Yes) indicated positive M stage. Tumor size was categorized into two groups: ≤3 cm and >3 cm because guideline proposed tumor size >3 was risk factor.^13^ With respect to regional nodes examined, according to the results of the K‐adaptive partitioning (KAPS) algorithm,^16^ the optimal cutoff was 0 and 4. Therefore, regional nodes examined was divided into three groups: 0, <=4, and >4. Primary site was recorded as head of pancreas, body of pancreas, tail of pancreas, pancreatic duct, and overlapping lesion/NOS. In our study, the main observation indicators were LNM status, overall survival (OS,) and cancer‐specific survival (CSS). CSS was defined as the length of time from either the date of diagnosis or the start of treatment for cancer to the date of death from cancer.

### 2.3 Statistical analysis

For the basic statistics, patients were divided into two groups, that is, 2004–2009 (internal validation) and 2010–2015 (external validation), and Pearson's chi‐squared test was utilized to investigate the association among the categorical variables. To explore the potential risk factors for LNM, we performed univariate and multivariate logical regression, and we presented the results as the odds ratio (OR) with the 95% confidence interval (CI). As for the analysis of LNM rate at different age, we performed the heat map to show the results. With respect to the OS and CSS of patients with IPMN, we performed survival curves using the survminer package in R software. Furthermore, to analyze the related risk factors for survival, we performed multivariate Cox regression, and we presented the result in the table. And also, we performed Lasso regression analysis to select the variables for constructing nomogram model. Finally, we selected CSS as the outcome of interest and performed nomogram based on the multivariate regression analysis. In addition, ROC curve, Calibration plot, and decision curve analysis (DCA) were performed to test the validity of the clinical predicting model we constructed.

All statistical analysis was performed with R software (version 3.6.1, StataCorp LLC). The main packages used in our study included ggplot2, survival, rms, pheatmap, kaps, ROCR, survminer, and glmnet package. The chi‐squared test was carried out with SPSS (version 24.0). The results were considered to be statistically significant when the P value was less than 0.05.

## 3 RESULTS

### 3.1 Baseline characteristics of enrolled patients with IPMN

As shown in Figure S1, we extracted 941 patients diagnosed in 2004–2015 according to the criteria of enrollment, which includes 454 patients in 2004–2009 and 487 patients in 2010–2015. Table S1 showed the characteristics of patients. The median survival of patients was 12 months, which was the similar between external and internal validation group. As for the basic features, we found patients with IPMN were more frequent to occur in older patient (>50 years old, 90%) and white race, while distribution of sex was no difference (53.13% vs. 46.87%). Additionally, lesions can be in different pathology grade, whereas they mainly located in the head of pancreas. Sadly, we found IPMN lesion were easy to occur LNM with a rate of 43.15% and major lesions were bigger than 3 cm. And many IPMNs were often in the advanced stage when was diagnosed (T3 stage: 51.01%).

### 3.2 Risk factors of LNM for patients with IPMN

**FIGURE 1:**
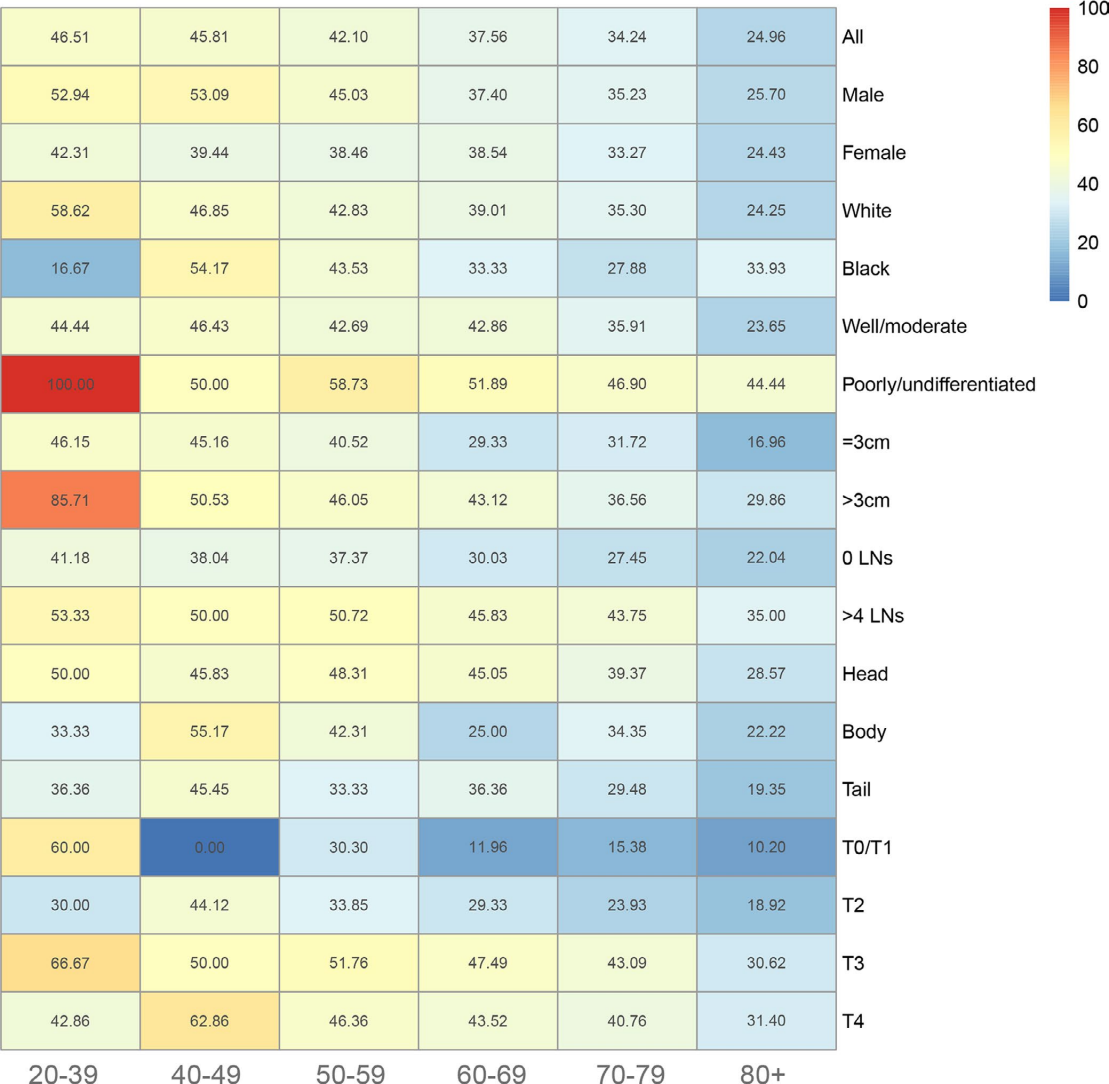
Heatmap showing rate of LNM of patients with IPMN among patients aged 20–39, 40–49, 50–59, 60–69, 70–79, and 80+ years stratified by different characteristics, respectively

To investigate the risk factors of LNM, we performed univariate and multivariate logistic regression analysis and found age, pathology grade, tumor size, regional nodes examined, and T stage were independent factors (Table 1). Of these factors, increased age were associated with lower risk of occurring LNM, for instance, compared with patients aged less than 50 years, patients aged >=70 had lower risk of LNM (OR, 0.528; 95%CI, 0.311–0.896; *p* = 0.018), while increased tumor size, advanced pathology grade, and T stage, and enhanced number of regional nodes examined were related with higher risk of LNM. To evaluate whether there was inverse association between age and LNM rate, we used 3470 patients with detailed information of LNM which were recorded in the Table S2 in 2004–2015, avoiding selecting bias during the process of analysis. As shown in the Figure 1, we found an inverse relationship was between LNM rate and age, the highest LNM rate was 46.51% in patients at 20–39 years old, which was deceased as the age increasing and decreased to 24.96% for patients aged 80+ years. And also, the analysis of linear trend suggested an increased age when diagnosed was correlated with lower risk of LNM (*p* = 0.0083) (Figure S2), which was in line with result of multivariate regression analysis. Subgroup analysis were performed to evaluate whether a similar trend existed in other groups stratified by sex, race, tumor size, pathology grade, tumor site, and T stage. Figure 1 showed patients aged 20–39 years had highest LNM rate while patients aged >=80 had the lowest LNM rate in most subgroups except some values were doubted.

**TABLE 1:** Univariate and Multivariate logistic regression analysis of clinical characteristics of IPMN patients for LNM

Variables	Univariate analysis		Multivariate analysis	
	OR (95%CI)	*p* value	OR (95%CI)	*p* value
Age		0.000		0.005
<50	Reference	—	Reference	—
50–70	0.881(0.542–1.432)	0.61	0.81(0.481–1.365)	0.429
>70	0.531(0.324–0.87)	0.012	0.528(0.311–0.896)	0.018
Race				
White	Reference	—		
Black	0.89(0.579–1.369)	0.596		
Other	0.643(0.412–1.002)	0.051		
Sex				
Female	Reference	—		
Male	0.78(0.602–1.01)	0.06		
Pathology Grade		0.000		0.004
Well	Reference	—	Reference	—
Moderately differentiated	1.701(1.23–2.354)	0.001	1.425(1.007–2.016)	0.046
Poorly	2.369(1.651–3.401)	0.000	2.055(1.394–3.029)	0.000
Undifferentiated	1.707(0.477–6.525)	0.434	1.501(0.374–6.024)	0.000
Tumor size				
≤3 cm	Reference	—	Reference	—
>3 cm	1.583(1.202–2.084)	0.001	1.365(0.992–1.878)	0.046
Regional nodes examined				0.000
0	Reference	—	Reference	—
<=4	1.383(0.85–2.25)	0.192	1.587(0.937–2.690)	0.086
>4	2.245(1.690–2.982)	0.000	2.563(1.836–3.579)	0.000
Primary site				
Head	Reference	‐		
Body	0.639(0.416–0.983)	0.041		
Tail	0.744(0.502–1.102)	0.14		
Pancreatic duct	0.696(0.250–1.1943)	0.49		
Overlapping lesion/NOS	0.807(0.553–1.177)	0.265		
T stage				0.000
T1	Reference	—	Reference	—
T2	2.188(1.154–4.151)	0.017	1.93(0.963–3.867)	0.064
T3	6.143(3.376–11.179)	0.000	4.403(2.306–8.408)	0.000
T4	4.448(2.311–8.561)	0.000	5.086(2.458–10.522)	0.000

### 3.3 Analysis of survival and its risk factors for patients with IPMN

With regard to the survival of patients, we performed K‐M survival analysis according to group (Figure S3). The result showed 1‐year, 3‐year, and 5‐year OS rate were 60%, 33%, and 21%, respectively (Figure S3A); similarly, 1‐year, 3‐year, and 5‐year CSS rate were 65%, 37% and 24%, respectively (Figure S3B). Additionally, we found there was significant difference between 2004–2009 and 2010–2015 year (*p* = 0.013). By the univariate and multivariate cox analysis (Table S3), age >70 year, T4 stage, N1 stage, M1 stage and poor differentiation were identified to be associated with poorer survival, while regional nodes examined were beneficial for patients. Moreover, K‐M survival analysis according to age suggested increased age predicted poorer prognosis (Figure S4A,B), which was opposite with LNM rate.

### 3.4 Constructing and validating nomogram for predicting LNM and survival externally and internally

**FIGURE 2:**
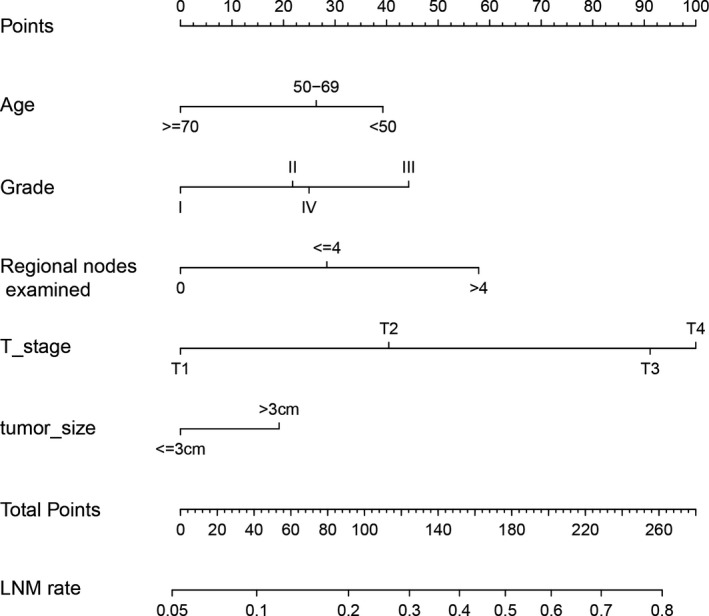
A nomogram was performed to predict LNM based on multivariate logistic regression analysis

**FIGURE 3:**
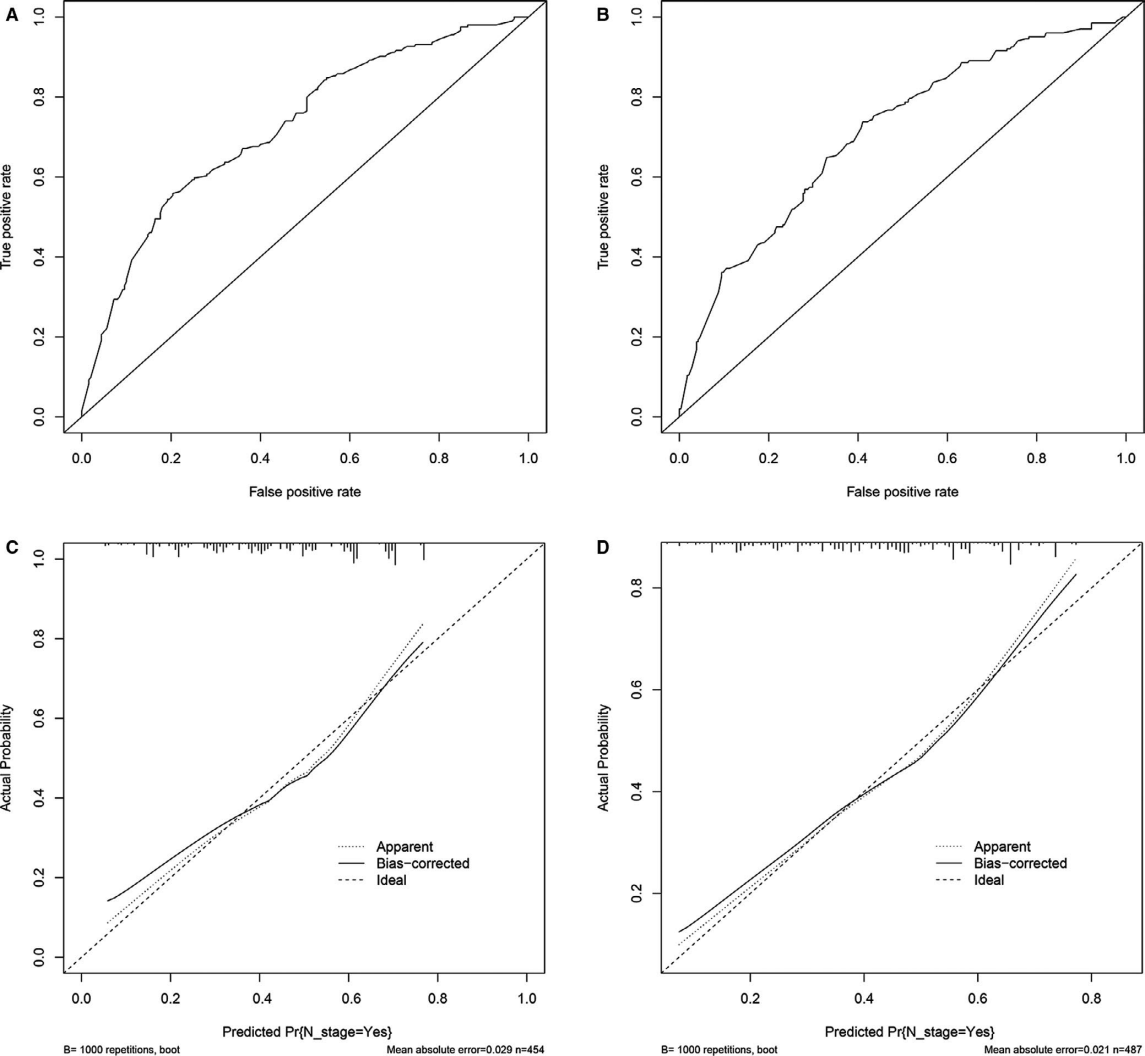
ROC curve and calibration curves of the nomogram to predict LNM in the internal and external cohort. A‐B, ROC curve of nomogram to predict LNM in the internal and external cohort. C‐D, The calibration curves of nomogram to predict LNM in the internal and external cohort

**FIGURE 4:**
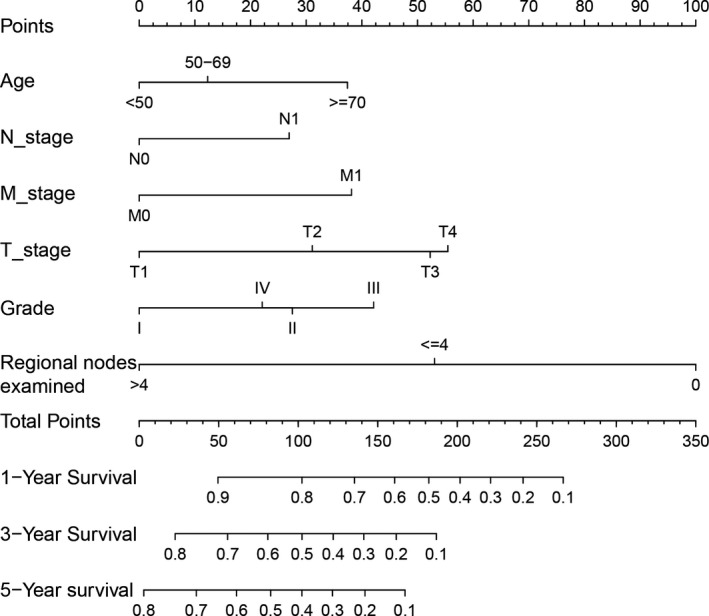
A nomogram was performed to predict survival based on multivariate cox and Lasso regression analysis

**FIGURE 5:**
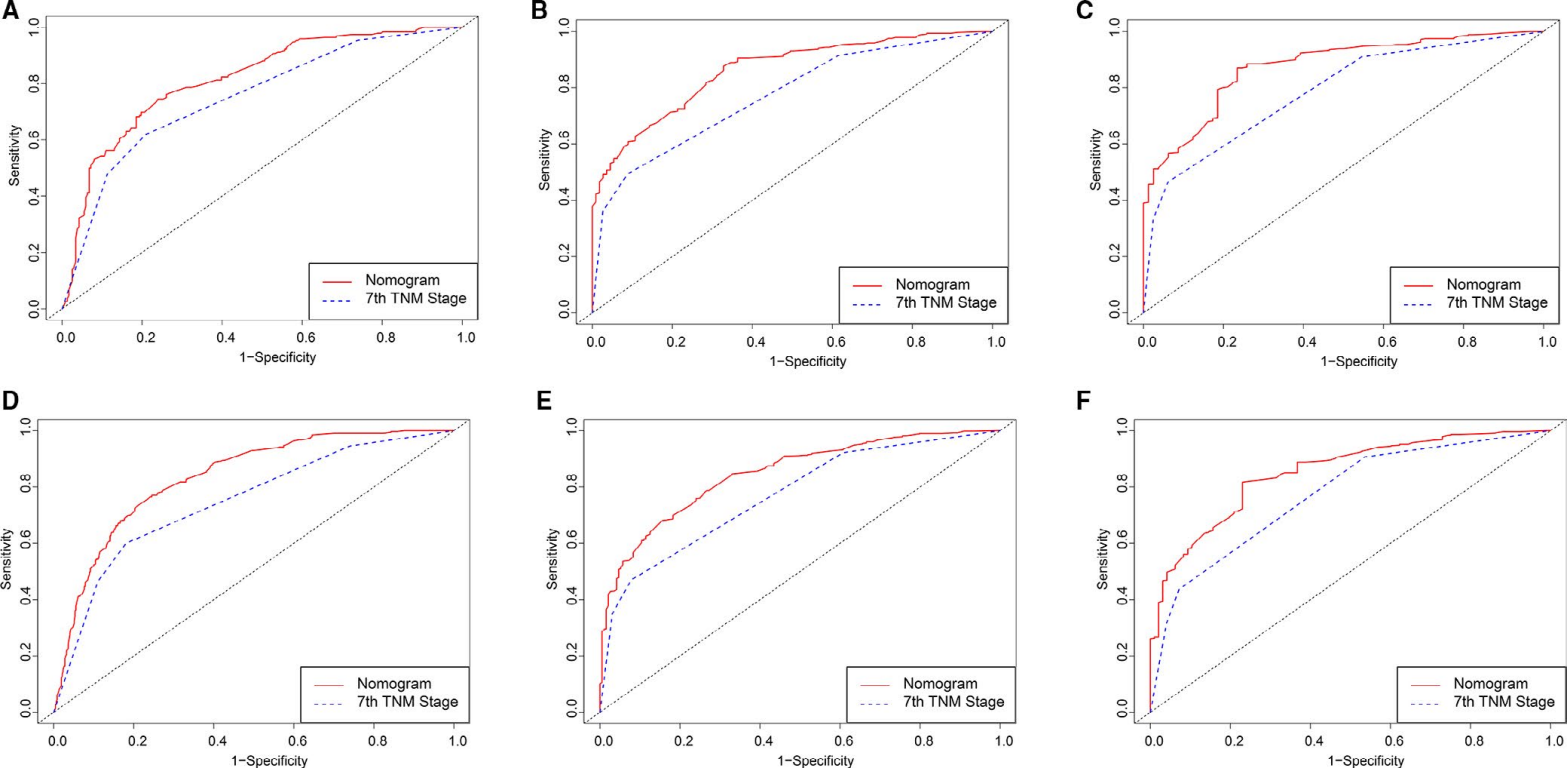
ROC curve of the Nomogram and 7th TNM Stage in prediction of prognosis of patients at 1, 3, and 5 year point in the 2004–2015 cohort. A‐C, 2004–2009 cohort (internal validation), A 1‐year survival predicting, B, 3‐year survival predicting, C, 5‐year survival predicting; D‐F 2010–2015 cohort (external validation), D, 1‐year survival predicting, E, 3‐year survival predicting, F 5‐year survival predicting

**FIGURE 6:**
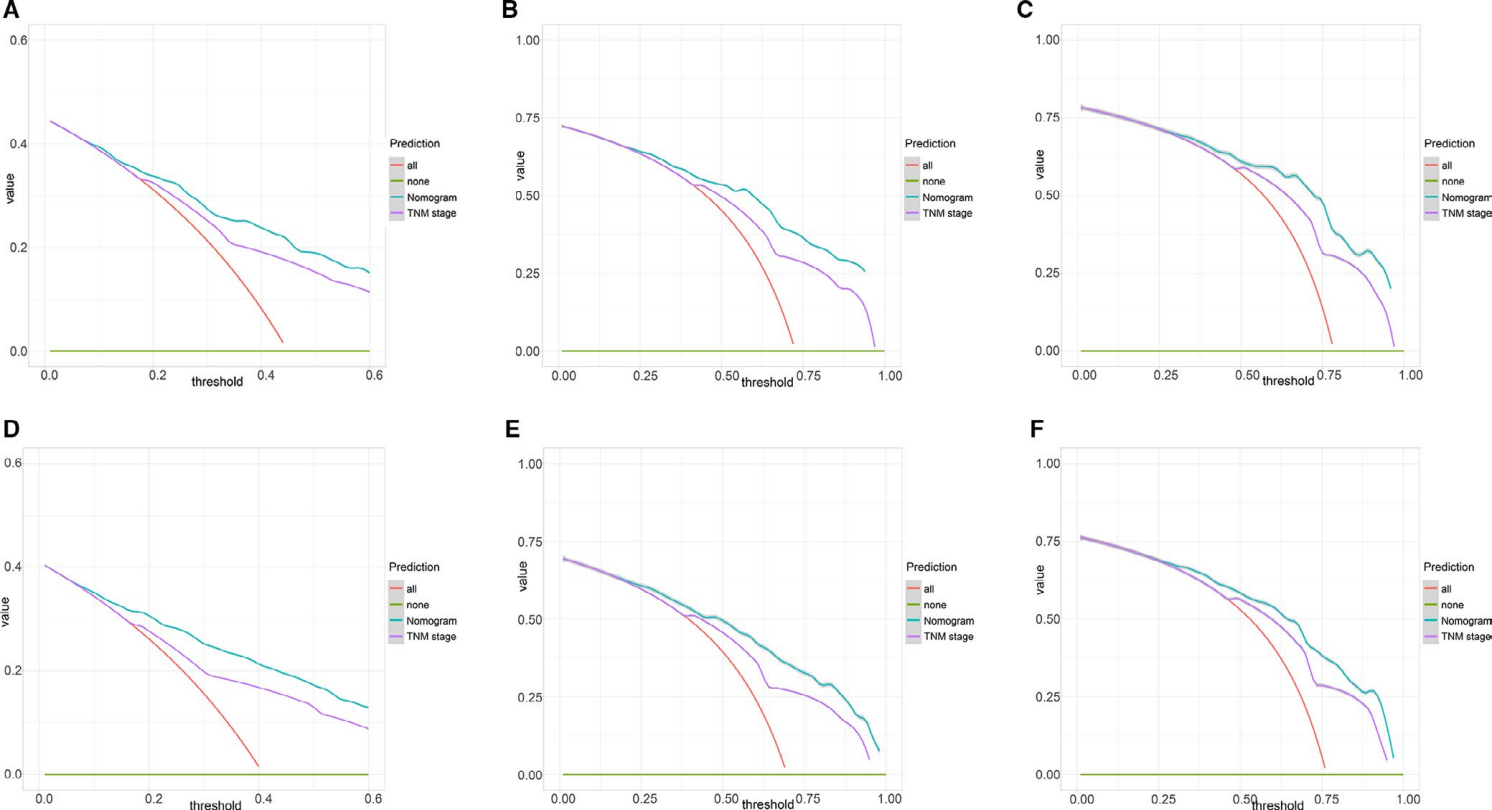
Decision curve analysis for the Nomogram and the model 7th TNM Stage in prediction of prognosis of patients at 1, 3‐, and 5‐year point in the internal validation and external validation group. A‐C, 1, 3‐, and 5‐year survival prediction in the internal cohort, respectively. D‐F, 1, 3‐, and 5‐year survival prediction in the external cohort, respectively

In term of LNM predicting model, we performed nomogram based on the multivariate logistic regression analysis. As shown in the Figure 2, we found T stage contributed the most to prognosis, followed by examined nodes, pathology grade, and age, while tumor size accounted for the least ratio. In the internal validation, the C‐indexes of the established nomogram to predict LNM was 0.735 (95%CI, 0.698–0.783) and bootstrap corrected value was 0.718 (Table S4). External validation showed similar predicting value with a C‐indexes of 0.741 (95%CI, 0.701–0.794, bootstrap corrected: 0.721) (Table S4). Furthermore, in the sensitivity and specificity analysis, the Area under ROC curve (AUC) in the internal and external group were 0.753 (95%CI, 0.711–0.821) and 0.761(95%CI, 0.715–0.831), respectively (Table S4 and Figure 3A,B). At the same time, we found no matter internal group or external group, good agreement was observed between the actual value and nomogram prediction (Figure 3C,D). To well establish survival predicting model, we first performed lasso regression analysis to select suitable variables for survival prediction. According to the result of lasso regression analysis (Figure S5), age, T stage, N stage, M stage, regional nodes examined and pathology grade were identified to be highly associated with survival, which was consistent with the result of multivariate cox regression analysis. Based on the result of lasso and cox regression analysis, nomogram was constructed and showed that regional nodes examined contributed the most to survival, followed by T stage, pathology grade, M stage, and age, while N stage was least effect (Figure 4). In the internal validation, the C‐indexes for nomogram to predict CSS was 0.768 (95%CI, 0.708–0.803) and bootstrap corrected value was 0.73, which was significantly higher than those of TNM stage (C‐indexes, 0.701; 95%CI, 0.683–0.736; bootstrap corrected: 0.686) (Table S5). Similarly, in the external validation, superiority of nomogram (0.771, 95%CI, 0.721–0.834) was also observed in comparison with TNM stage (0.695, 95%CI, 0.651–0.729) (Table S5). In addition, as for the analysis of sensitivity and specificity of predicting CSS, nomogram also performed better than TNM stage both in the internal validation group (1‐year AUC:0.753 vs. 0.693, 3‐year AUC: 0.801 vs. 0.731, 5‐year AUC: 0.803 vs. 0.733, *p* < 0.05) (Figure 5A–C and Table S5) and external validation group (1‐year AUC: 0.761 vs. 0.701, 3‐year AUC: 0.772 vs. 0.713, 5‐year AUC:0.811 vs. 0.735, *p* < 0.05) (Figure 5D–F and Table S5). Finally, to compare the clinical usability between nomogram and TNM stage, we performed DCA and showed results in the Figure 6. For predicting 1‐year, 3‐year, and 5‐year CSS, no matter in the internal cohort or external cohort, the nomogram showed a greater benefit across the period of follow‐up compared to TNM stage.

## 4 DISCUSSION

With the improvement of devices including endoscopic ultrasound (EUS) and fine‐needle aspiration biopsy for diagnosis of IPMN, this disease has increased over the decades.^17^ As for the criteria for surgery and surveillance, what several guidelines defined was controversial, most of which took patients status and performance of imaging into account when determining the surgery.^4^ However, whether patients need chemotherapy or undergo surgery alone, there is no detailed criteria to use. In our study, we analyzed the survival of patients after surgery without chemotherapy and build a nomogram to predict prognosis. We found the survival rate of 5‐year is not up to 30% and lymphadenectomy (>4 nodes) does much benefit for patients. In addition, we found IPMN was easy to occur LNM, especially for advanced T stage and poor differentiated (Figure 1). The nomogram we established to predict LNM had a high predicting efficacy with a C‐index value of 0.735 and an AUC of 0.753. Interestingly, including T1 stage, we found an inverse correlation was between age and LNM.

As for the survival of IPMN, it depends on the malignance. A large population study found patients with IPMN only had a median survival of 28.9 months, 1‐year survival rate of 76% and 5‐year survival rate of 17%, which was in line with our results.^18^ Some studies reported 5‐year survival rate would be 35–50% when performed surgery alone, while preoperative or postoperative adjuvant chemotherapy were able to prolong survival with a 5‐year survival rate of 50–80%, especially for lesions with positive LNM.^19^, ^20^ That is to say, clinically, our doctors are not usually well to evaluate the invasion of IPMN, leading to inaccurate judgment for the disease progression. A cohort study with 286 patients found the accuracy of international consensus standard in 2012 for invasive IPMN is only 69%.^21^ LNM was considered to be an important feature of invasive IPMN, a study with 286 patients showed the LNM rate of patients with IPMN was 36.5% or so, which was lower compared to our result (46.51%).^21^ However, some study found LNM rate for invasive IPMN was 37.5%–45%, which was similar with our findings.^22^, ^23^ In our nomogram to predict LNM, the C‐index and Area under ROC curve was 0.735 and 0.753, respectively, suggesting it was effective to use. According to the high‐risk stigmata proposed by guidelines in 2012, LNM was a part of high‐risk stigmata and had high preoperative diagnosis ability, additionally, for those lesion with LNM, pancreatectomy with lymphadenectomy was necessary instead of limited surgery.^24^ To our knowledge, it is the first time to perform comprehensive analysis of LNM, moreover, we found inverse association was between age and LNM. The LNM rate of patients aged 20–39 year was 46.51%, while LNM rate aged >=80 years was 24.96% which was far lower than that of 20–39 years patients. Similarly, some tumors showed the same results, such as colorectal cancer and gastric cancer.^25^, ^26^ The increased risk to occur LNM for younger patients suggested it ought to be carefully and pay more attention for younger patients during the process of making decisions. However, the number of patients aged 20–49 years were only 198, to some extent, which reduced the credibility of the result. Hence, it remained to need a study based on larger population.

Traditionally, to decide to monitor patients with IPMN was usually based on age, history of family, clinical symptoms, and risk of progressing into cancer, which was a tackle problem for clinical judgment.^27^ In fact, according to different guidelines, there remained to be controversy about how to perform surveillance for IPMN patients.^24^, ^28^ To our knowledge, for predicting the survival of IPMN, a similar nomogram was established and demonstrated effectively compared with 7^th^ TNM stage (C‐indexes, 0.756 vs. 0.645).^29^ Compared with our model, the nomogram created earlier was unreasonable for the parameters selection, while our model was reasonable and logic by lasso regression analysis which can prevent over fitting of the model and multivariate cox regression analysis that ensure the accuracy of model.^30^ In our model, we recruited age, pathology grade, and examined lymph nodes other than TNM stage, avoiding the insufficiency of TNM stage. Naturally, nomogram we conducted performed better than TNM stage, which was tested by ROC curve and DCA analysis (Figures 5 and 6). However, the disadvantage is that we use 7^th^ TNM stage rather than 8^th^ TNM stage, therefore, which is better between nomogram and 8^th^ TNM stage needs further study. In addition, our nomogram was constructed based on CSS indicator instead of other indexes such as recurrence rate and disease‐free survival, resulting in limited clinical value of our model.

Surgery is recommended for all mucinous neoplasms and main duct neoplasms.^6^ For patients with invasive ductal adenocarcinoma of the pancreas, postresection adjuvant therapy improves survival, even in patients with positive margins or involved lymph nodes.^6^ There is controversy as to the best adjuvant strategy.^31^ Due to the significant morbidity and mortality associated with pancreaticoduodenectomy or distal pancreatectomy, the patient's and surgeon's decision to perform surgery should include factors such as the patient's age and general health, the malignant risk of the lesion, and the suspicion for malignancy.^21^ Our nomogram model was performed by combining several clinical characteristics and was proved to be highly effective to predict lymph node metastasis. Several clinical techniques such as microforces biopsy and endoscopic ultrasound was a little restricted. Our model would improve the diagnostic accuracy and clinical management, for instance, adjuvant chemotherapy or radiotherapy was associated with significant improved overall survival only in presence of nodal metastasis.^32^ That is to say, our findings would help clinicians to select adjuvant treatment for patient with potential risk of LNM. Similarly, our nomogram model predicting prognosis also has some advantages compared to other traditional criteria. First of all, nomograms are widely used to assess the prognosis of cancers because of their ability to transform a statistical predictive model into a single numerical estimate of the probability of an event, which is a user‐friendly method that guides clinical decision‐making for doctors.^14^ Secondly, our model was more accurate to assess prognosis of patients compared to traditional TNM staging. As we know, surveillance is resource‐consuming and must be balanced against the likely benefits and perceived risks for malignant transformation. Therefore, our model predicting LNM and survival was useful.

In conclusion, we used SEER data of patients with IPMN, performed multivariate logistic analysis, and constructed a nomogram to predict LNM with a C‐index value of 0.768. Additionally, we observed an inverse association between age and LNM, which suggested early‐onset had higher risk to be invasive IPMN. As for the survival predicting model, nomogram showed a better clinical application value compared to 7^th^ TNM stage, which was demonstrated by ROC curve and DCA analysis. Our study could help doctors to make judgment for surgery, however, the interpretation should be with caution and another cohort study needs to validate our findings.

## 5 ETHICS STATEMENT

Ethics approval and consent was obtained from SEER database.

## CONFLICTS OF INTEREST

The authors disclose no conflicts.

## AUTHOR CONTRIBUTIONS

CTT and Peng Wang: data collection, data analysis, and manuscript writing. BXL: data analysis. CYZ and YXC: project development.

## Supporting information

Fig S1

Fig S2

Fig S3

Fig S4

Fig S5

Table S1

Table S2

Table S3

Table S4

Table S5

Supplementary Material
