# Supplementary figures and images for: Analyzing and predicting the LNM rate and prognosis of patients with intraductal papillary mucinous neoplasm of the pancreas
Source: Cancer Med. 2021 Feb 27;10(6):1925–35. doi: 10.1002/cam4.3632 (PMC7957210; doi:10.1002/cam4.3632)

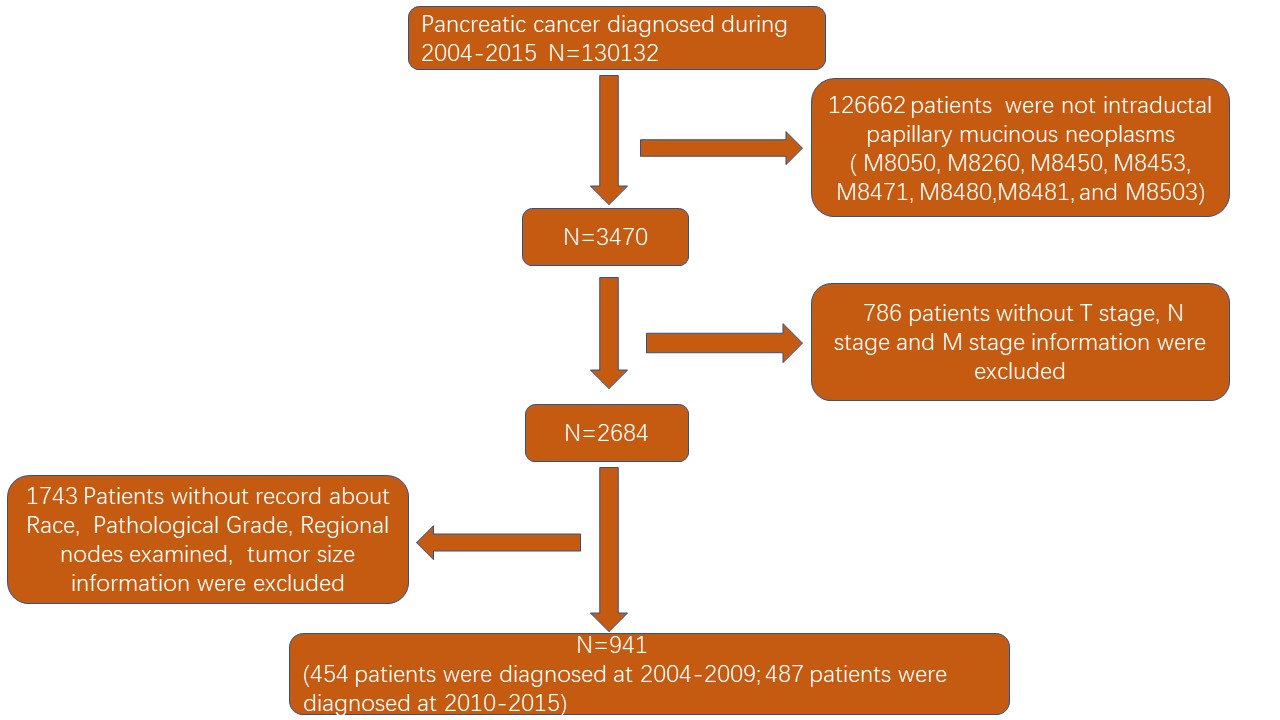

Supplement: Supplementary file 1 — Fig S1 [file CAM4-10-1925-s001.jpg]

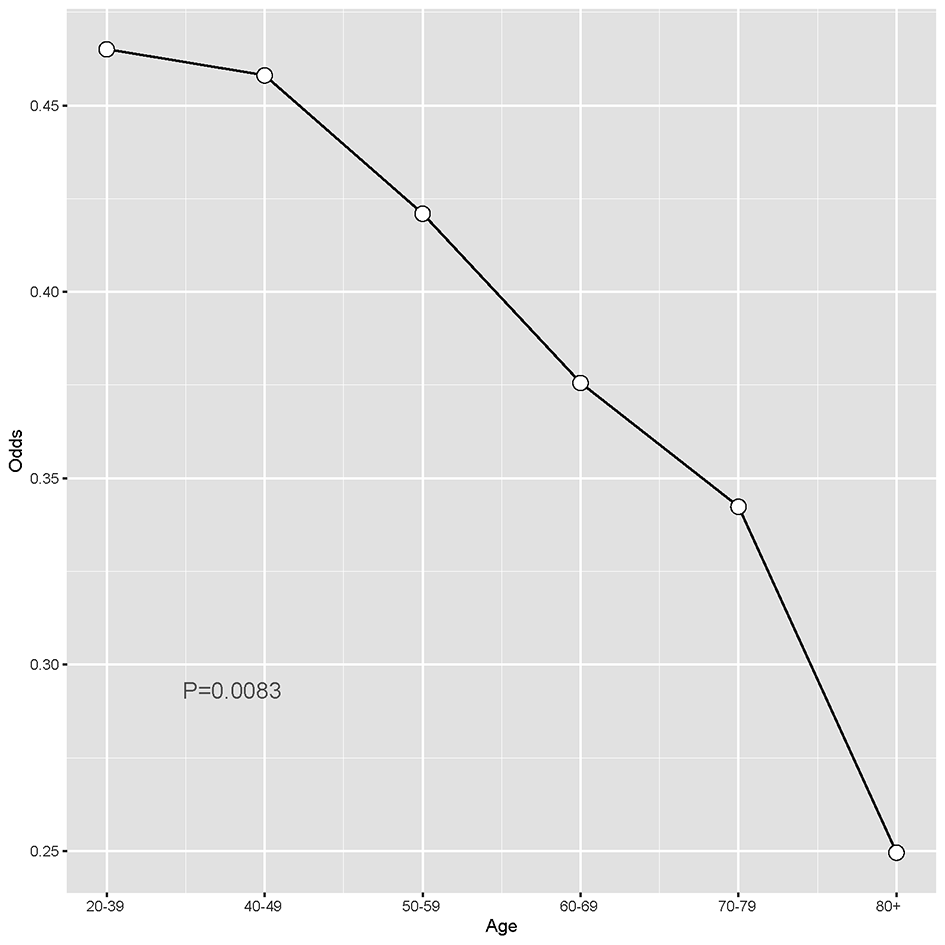

Supplement: Supplementary file 2 — Fig S2 [file CAM4-10-1925-s002.tif]

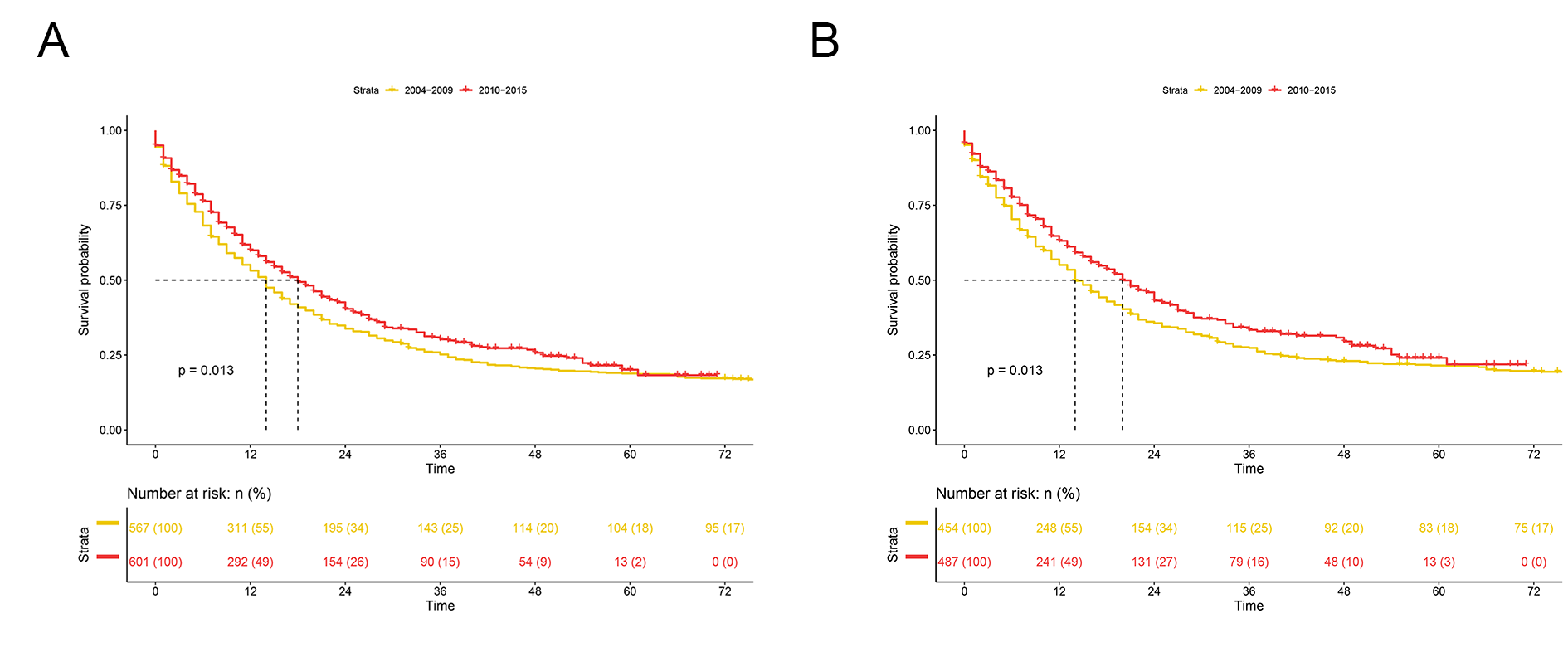

Supplement: Supplementary file 3 — Fig S3 [file CAM4-10-1925-s010.tif]

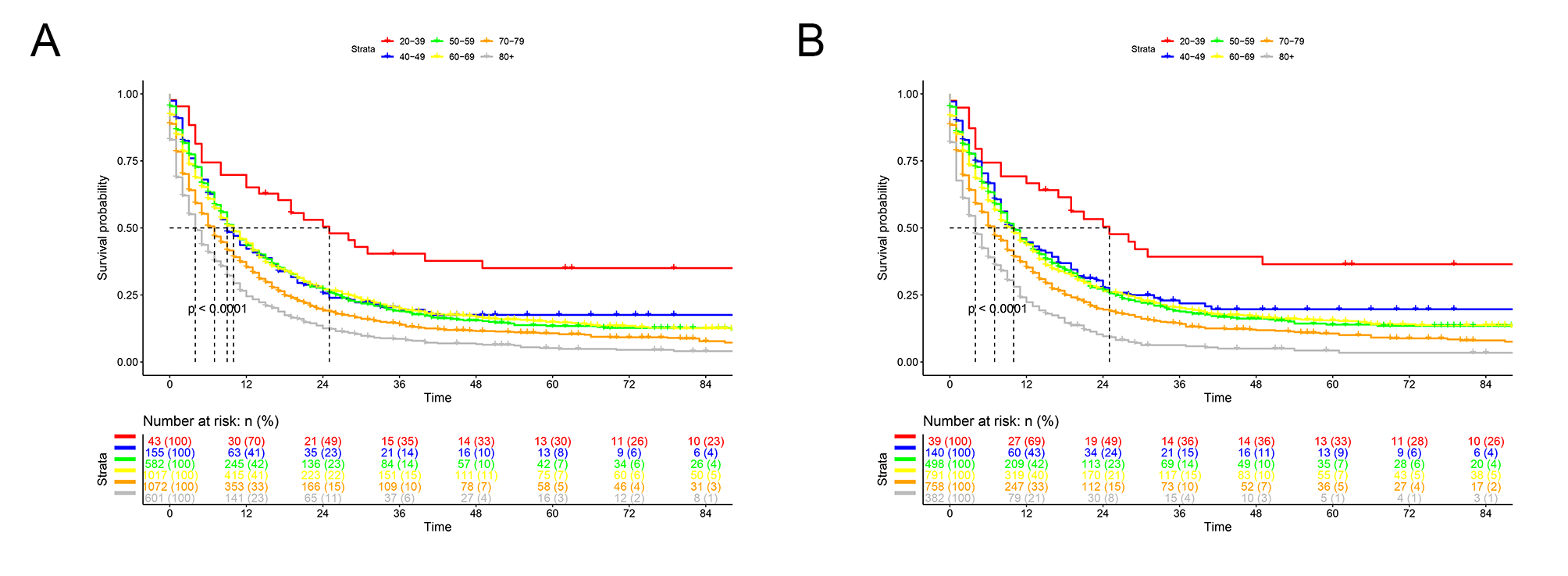

Supplement: Supplementary file 4 — Fig S4 [file CAM4-10-1925-s003.tif]

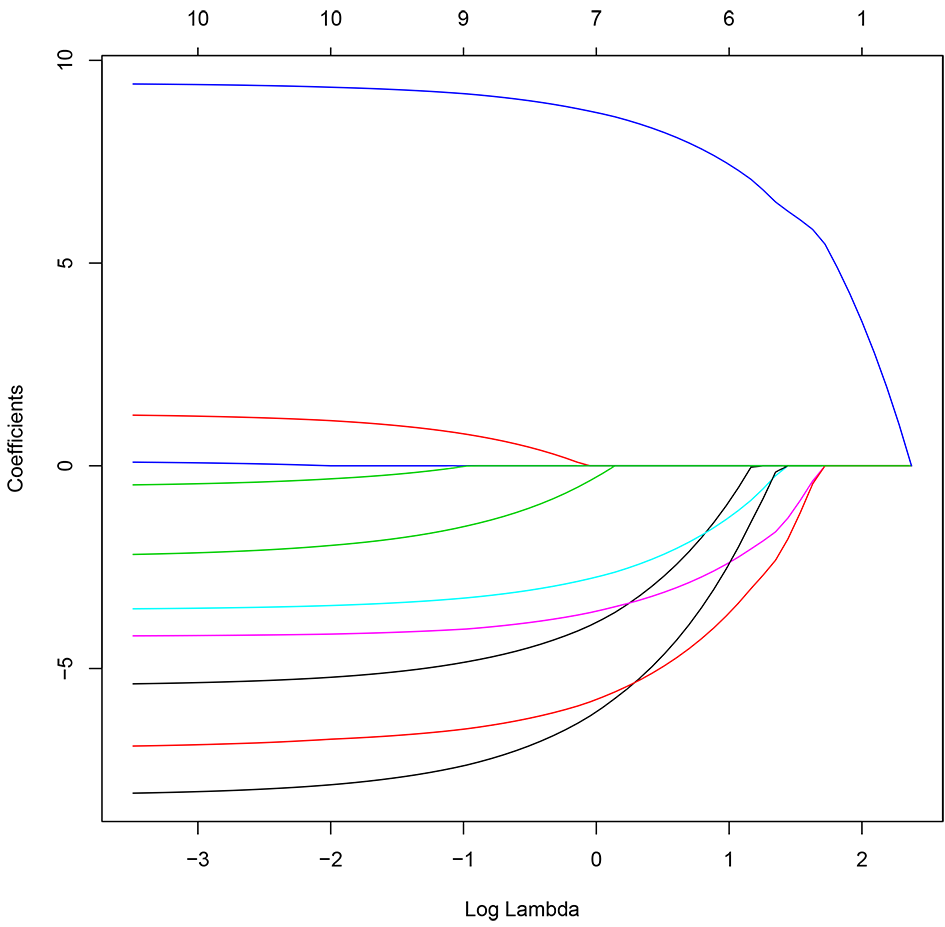

Supplement: Supplementary file 5 — Fig S5 [file CAM4-10-1925-s009.tif]
