# Supplementary material for: Analyzing and predicting the LNM rate and prognosis of patients with intraductal papillary mucinous neoplasm of the pancreas
Source: Cancer Med. 2021 Feb 27;10(6):1925–35. doi: 10.1002/cam4.3632 (PMC7957210; doi:10.1002/cam4.3632)
Supplement: Supplementary file 6 — Table S1 [file CAM4-10-1925-s006.docx]

**Supplementary Table 1: Patients’ demographics, clinical characteristics at diagnosis**

| Variables | Total (%) | 2004-2009 | 2010-2015 | P Value |
| --- | --- | --- | --- | --- |
| n | 941 | 454(48.25%) | 487(51.75%) |  |
| Age |  |  |  | 0.919 |
| <50 | 76(8.08%) | 36(7.93%) | 40(8.21%) |  |
| 50-70 | 461(48.99%) | 220(48.46%) | 241(49.49%) |  |
| >70 | 404(42，93%) | 198(43.61%) | 206(42.30%) |  |
| Race |  |  |  | 0.104 |
| White | 748(79.49%) | 371(81.72%) | 377(77.41%) |  |
| Black | 96(10.20%) | 46(10.13%) | 50(10.27%) |  |
| Other | 97(10.31%) | 37(8.15%) | 60(12.32%) |  |
| Sex |  |  |  | 0.109 |
| Female | 500(53.13%) | 229(50.44%) | 271(55.65%) |  |
| Male | 441(46.87%) | 225(49.56%) | 216(44.35%) |  |
| Pathology Grade |  |  |  | 0.184 |
| Well | 257(27.31%) | 120(26.43%) | 137(28.13%) |  |
| Moderately differentiated | 426(45.27%) | 197(43.39%) | 229(47.02%) |  |
| Poorly | 249(26.46%) | 134(29.52%) | 115(23.61%) |  |
| Undifferentiated | 9(0.96%) | 3(0.66%) | 6(1.23%) |  |
| Lymph node Metastasis |  |  |  | 0.284 |
| NO | 535(56.85%) | 250(55.07 %) | 285(58.52%) |  |
| Yes | 406(43.15%) | 204(44.93%) | 202(41.48%) |  |
| Metastasis |  |  |  | 0.192 |
| No | 702(74.60%) | 330(72.69%) | 372(76.39%) |  |
| Yes | 239(25.40%) | 124(27.31%) | 115(23.61%) |  |
| Tumor size |  |  |  | 0.045 |
| ≤3cm | 331(35.18%) | 145(31.94%) | 186(38.19%) |  |
| >3cm | 610(64.82%) | 309(68.06%) | 301(61.8%) |  |
| Regional_nodes_examined |  |  |  | 0.271 |
| 0 | 352(37.41%) | 175(38.55%) | 177(36.34%) |  |
| <=4 | 96(9.14%) | 52(11.45%) | 44(9.03%) |  |
| >4 | 493(53.45%) | 227(50.00%) | 266(54.63%) |  |
| Primary site |  |  |  | 0.2562 |
| Head | 551(58.55%) | 275(60.57%) | 276(56.67%) |  |
| Body | 107(11.37%) | 46(10.13%) | 61(12.53%) |  |
| Tail | 128(13.60%) | 57(12.56%) | 71(14.58%) |  |
| Pancreatic duct | 16(1.70%) | 11(2.42%) | 5(1.03%) |  |
| Overlapping lesion/NOS | 139(14.77%) | 65(14.32%) | 74(15.20%) |  |
| T stage |  |  |  | 0.0009 |
| T1 | 88(9.35%) | 35(7.71%) | 53(10.88%) |  |
| T2 | 222(23.59%) | 115(25.33%) | 107(21.97%) |  |
| T3 | 480(51.01%) | 212(46.70%) | 268(55.03%) |  |
| T4 | 151(16.05%) | 92(20.26%) | 59(12.11%) |  |
| 7th TNM stage |  |  |  | 0.07 |
| I | 166(17.64%) | 76(16.74%) | 90(18.48%) |  |
| II | 449(47.72%) | 203(44.71%) | 246(50.51%) |  |
| III | 87(9.25%) | 51(11.23%) | 36(7.39%) |  |
| IV | 239(25.40%) | 124(27.31%) | 115(23.61%) |  |
| Median survival (quartile,M) | 12(5-143) | 12(5,143) | 12(5-139) |  |
