# Supplementary material for: Analyzing and predicting the LNM rate and prognosis of patients with intraductal papillary mucinous neoplasm of the pancreas
Source: Cancer Med. 2021 Feb 27;10(6):1925–35. doi: 10.1002/cam4.3632 (PMC7957210; doi:10.1002/cam4.3632)
Supplement: Supplementary file 7 — Table S2 [file CAM4-10-1925-s008.docx]

**Supplementary Table 2. Baseline characteristics of patients with IPMN by age at diagnosis**

| Variable | Total | Age at dignosis, n(%) | | | | | |
| --- | --- | --- | --- | --- | --- | --- | --- |
|  |  | 20-39 | 40-49 | 50-59 | 60-69 | 70-79 | 80+ |
| Total | 3470 | 43  (1.24%) | 155  (4.47%) | 582  (16.77%) | 1017  (29.3%) | 1072  (30.89%) | 601  (17.32%) |
| Sex |  |  |  |  |  |  |  |
| Male | 1721 | 17  (39.5%) | 81  (52.2%) | 322  (55.3%) | 524  (51.5%) | 528  (49.3%) | 249  (41.4%) |
| Female | 1749 | 26  (60.5%) | 74  (47.7%) | 260  (44.7%) | 493  (48.5%) | 544  (50.7% | 352  (58.6%) |
| Race |  |  |  |  |  |  |  |
| White | 2787 | 29  (67.4%) | 111  (71.6%) | 453  (77.8%) | 810  79.6%) | 881  (82.2%) | 503  (83.7%) |
| Black | 386 | 6  (13.9%) | 24  (15.5% | 85  (14.6%) | 111  (10.9%) | 104  (9.7%) | 56  (9.32%) |
| Other | 286 | 8  (18.6%) | 20  (12.9%) | 40  (6.87%) | 93  (9.14%) | 85  (7.93%) | 40  (6.65%) |
| Unknown | 11 | 0 | 0 | 4  (0.69%) | 3  (0.3%) | 2  (0.2%) | 2  (0.33%) |
| Pathology Grade |  |  |  |  |  |  |  |
| Well | 383 | 7  (16.3%) | 21  (13.5%) | 63  (10.8%) | 104  (10.2%) | 121  (11.2%) | 67  (11.1%) |
| Moderately differentiated | 654 | 11  (25.6%) | 34  (21.9%) | 108  (18.6%) | 218  (21.4%) | 202  (18.8%) | 81  (13.5%) |
| Poorly | 364 | 2  (4.7%) | 15  (9.7%) | 59  (10.1%) | 104  (10.2%) | 139  (13%) | 45  (7.5%) |
| Undifferentiated | 13 | 0 | 1  (0.64%) | 4  (0.7%) | 2  (0.2%) | 6  (0.6%) | 0 |
| Unknown | 2056 | 23  (53.5%) | 84  (54.2%) | 348  (59.8%) | 589  (57.9%) | 604  (56.3%) | 408  (67.9%) |
| Lymph node Metastasis |  |  |  |  |  |  |  |
| NO | 2231 | 23  (53.5%) | 84  (54.2%) | 337  (57.9%) | 631  (62%) | 705  (65.8%) | 451  (75%) |
| Yes | 1239 | 20  (46.5%) | 71  (45.8%) | 245  (42.1%) | 386  (38%) | 367  (34.2%) | 150  (25%) |
| Tumor size |  |  |  |  |  |  |  |
| ≤3cm | 958 | 13  (30.2%) | 31  (20%) | 153  (26.3%) | 300  (29.5%) | 290  (27%) | 171  (28.5%) |
| >3cm | 1841 | 21  (48.8%) | 95  (61.3%) | 304  (52.2%) | 545  (53.6%) | 588  (54.9%) | 288  (47.9%) |
| Unknown | 671 | 9  (20.9%) | 29  (18.7%) | 125  (21.5%) | 172  (16.9%) | 194  (18.1%) | 142  (23.6%) |
| Regional_nodes_examined |  |  |  |  |  |  |  |
| 0 | 2279 | 17  (39.5%) | 92  (59.4%) | 388  (66.7%) | 616  (60.6%) | 685  (63.9%) | 481  (80%) |
| <=4 | 233 | 5  (11.6%) | 17  (39.5%) | 34  (5.8%) | 71  (7%) | 79  (7.4%) | 27  (4.5%) |
| >4 | 760 | 14  (32.6%) | 33  (76.7%) | 126  (21.6%) | 256  (25.2%) | 251  (23.4%) | 80  (13.3%) |
| Unknown | 123 | 6  (14%) | 4  (9.3%) | 22  (3.8%) | 42  (4.1%) | 36  (3.4%) | 13  (2.2%) |
| Primary site |  |  |  |  |  |  |  |
| Head | 1635 | 18  (41.9%) | 72  (46.5%) | 236  (40.5%) | 495  (48.7%) | 541  (50.5%) | 273  (45.4%) |
| Body | 462 | 3  (7%) | 29  (18.7%) | 78  (13.4%) | 140  (13.8%) | 131  (12.2%) | 81  (13.5%) |
| Tail | 537 | 11  (25.6%) | 22  (14.2%) | 106  (18.2%) | 154  (15.1%) | 162  (15.11%) | 82  (13.6%) |
| Pancreatic duct | 48 | 0 | 2  (4.7%） | 11  (1.9%) | 12  (1.2%) | 12  (1.1%) | 11  (1.8%) |
| Overlapping lesion/NOS | 789 | 11  (25.7%) | 30  (19.4%0 | 151  (25.9%) | 216  (21.2%) | 227  (21.2%) | 154  (25.6%) |
| T stage |  |  |  |  |  |  |  |
| T1 | 264 | 5  (11.6%) | 7  (16.3%) | 33  (5.7%) | 92  (9.%) | 78  (7.3%) | 49  (4.6%) |
| T2 | 766 | 12  (27.9%) | 34  (21.9%) | 130  (22.3%) | 208  (20.4%) | 234  (21.8%) | 148  (24.6%) |
| T3 | 1302 | 16  (37.2%) | 62  (40%) | 199  (34.2%) | 389  (38.2%) | 427  (39.8%) | 209  (34.8%) |
| T4 | 615 | 7  (16.3%) | 35  (22.6%) | 110  (18.9%) | 193  (19%) | 184  (17.2%) | 86  (14.3%) |
| Unknown | 523 | 3  (7%) | 17  (11%) | 110  (18.9%) | 135  (13.3%) | 149  (13.9%) | 109  (18.1%) |
