# Supplementary material for: Analyzing and predicting the LNM rate and prognosis of patients with intraductal papillary mucinous neoplasm of the pancreas
Source: Cancer Med. 2021 Feb 27;10(6):1925–35. doi: 10.1002/cam4.3632 (PMC7957210; doi:10.1002/cam4.3632)
Supplement: Supplementary file 8 — Table S3 [file CAM4-10-1925-s011.docx]

**Supplementary Table 3: Univariate and multivariate Cox analysis of clinical characteristics for prognosis of IPMN for CSS.**

| Variables | Univariate analysis | | Multivariate analysis | |
| --- | --- | --- | --- | --- |
|  | HR (95%CI) | P  value | HR (95%CI) | P Value |
| Age |  | 0.000 |  | 0.000 |
| <50 | Reference | - | Reference | - |
| 50-70 | 1.105(0.812-1.504) | 0.525 | 1.188(0.864-1.636) | 0.289 |
| >70 | 1.508(1.106-2.054) | 0.009 | 1.63(1.182-2.247) | 0.003 |
| Race |  | 0.024 |  | 0.283 |
| White | Reference | - | Reference | - |
| Black | 1.095(0.843-1.423) | 0.498 | 1.019(0.799-1.334) | 0.889 |
| Other | 0.684(0.512-0.915) | 0.011 | 0.788(0.584-1.063) | 0.118 |
| Sex |  | 0.014 |  | 0.095 |
| Female | Reference | - | Reference | - |
| Male | 1.217(1.040-1.425) | 0.014 | 1.15(0.976-1.356) | 0.095 |
| Pathology Grade |  |  |  |  |
| Well | Reference | - | Reference | - |
| Moderately differentiated | 1.427(1.165-1.748) | 0.001 | 1.491(1.207-1.842) | 0.000 |
| Poorly | 1.995(1.596-2.494) | 0.000 | 1.787(1.417-2.253) | 0.000 |
| Undifferentiated | 1.422(0.628-3.223) | 0.399 | 1.306(0.571-2.987) | 0.528 |
| Lymph node Metastasis |  |  |  |  |
| NO | Reference | - | Reference | - |
| Yes | 1.363(1.163-1.597) | 0.000 | 1.472(1.237-1.751) | 0.000 |
| Metastasis |  |  |  | 0.000 |
| No | Reference | - | Reference | - |
| Yes | 3.526(2.961-4.2) | 0.000 | 1.707(1.376-2.117) | 0.000 |
| Tumor size |  |  |  |  |
| ≤3cm | Reference |  | Reference | - |
| >3cm | 1.504(1.269-1.781) | 0.000 | 1.090(0.906-1.312) | 0.359 |
| Regional_nodes_examined |  | 0.000 |  |  |
| 0 | Reference | - | Reference | - |
| <=4 | 0.439(0.335-0.576) | 0.000 | 0.512(0.38-0.689) | 0.000 |
| >4 | 0.225(0.189-0.268) | 0.000 | 0.247(0.196-0.311) | 0.000 |
| Primary site |  | 0.001 |  | 0.307 |
| Head | Reference | - | Reference | - |
| Body | 1.622(1.269-2.073) | 0.000 | 1.118(0.862-1.45) | 0.399 |
| Tail | 1.271(1.006-1.606) | 0.045 | 0.989(0.769-.270) | 0.938 |
| Pancreatic duct | 0.886(0.47201.661) | 0.705 | 1.89(0.983-3.635) | 0.056 |
| Overlapping lesion/NOS | 1.278(1.019-1.604) | 0.034 | 0.956(0.751-1.217) | 0.715 |
| T stage |  | 0.000 |  | 0.000 |
| T1 | Reference | - | Reference | - |
| T2 | 1.982(1.371-2.866) | 0.000 | 1.448(0.971-2.158) | 0.069 |
| T3 | 2.212(1.566-3.124) | 0.000 | 2.015(1.378-2.947) | 0.000 |
| T4 | 4.782(3.298-6.934) | 0.000 | 1.979(1.307-2.997) | 0.001 |
