# Supplementary material for: Analyzing and predicting the LNM rate and prognosis of patients with intraductal papillary mucinous neoplasm of the pancreas
Source: Cancer Med. 2021 Feb 27;10(6):1925–35. doi: 10.1002/cam4.3632 (PMC7957210; doi:10.1002/cam4.3632)
Supplement: Supplementary file 9 — Table S4 [file CAM4-10-1925-s007.docx]

**Supplementary Table 4:** **Accuracy of the prediction score of the nomogram for estimating the Risk of LNM Presence**

| Variable | Value (95%CI) | |
| --- | --- | --- |
|  | Internal validation | External validation |
| C index | 0.735 (0.698-0.783) | 0.741 (0.701-0.794) |
| C index  (bootstrap corrected) | 0.718 | 0.721 |
| Area under ROC curve | 0.753(0.711-0.821) | 0.761(0.715-0.831) |
| Sensitivity,% | 72.1(67.2-76.3) | 69.4(60.9-75.8) |
| Specificity,% | 71.9(68.3-75.8) | 80.1(73.7-86.3) |
