# Supplementary material for: Analyzing and predicting the LNM rate and prognosis of patients with intraductal papillary mucinous neoplasm of the pancreas
Source: Cancer Med. 2021 Feb 27;10(6):1925–35. doi: 10.1002/cam4.3632 (PMC7957210; doi:10.1002/cam4.3632)
Supplement: Supplementary file 10 — Table S5 [file CAM4-10-1925-s004.docx]

**Supplementary Table 5:** **Accuracy of the prediction score of the nomogram and TNM stage for estimating prognosis of patients with IPMN**

| Variable | Value (95%CI) | |
| --- | --- | --- |
|  | Internal validation | External validation |
| C index for nomogram | 0.768 (0.708-0.803) | 0.771 (0.721-0.834) |
| C index  (bootstrap corrected) | 0.73 | 0.75 |
| C index for TNM stage | 0.701(0.683-0.736) | 0.695(0.651-0.729) |
| C index  (bootstrap corrected) | 0.686 | 0.667 |
| 1 year AUC for nomogram | 0.753(0.711-0.821) | 0.761(0.715-0.831) |
| 3 year AUC for nomogram | 0.801(0.762-0.863) | 0.773 (0.659-0.828) |
| 5 year AUC for nomogram | 0.803(0.753-0.868) | 0.811 (0.703-0.847) |
| 1 year AUC for TNM stage | 0.693(0.671-0.801) | 0.701(0.685-0.795) |
| 3 year AUC for TNM stage | 0.731(0.701-0.804) | 0.713 (0.675-0.778) |
| 5 year AUC for TNM stage | 0.733(0.753-0.868) | 0.735 (0.683-0.797) |
