# Supplementary material for: Analyzing and predicting the LNM rate and prognosis of patients with intraductal papillary mucinous neoplasm of the pancreas
Source: Cancer Med. 2021 Feb 27;10(6):1925–35. doi: 10.1002/cam4.3632 (PMC7957210; doi:10.1002/cam4.3632)
Supplement: Supplementary file 11 — Supplementary Material [file CAM4-10-1925-s005.docx]

**Supplementary Figure 1:** The flow chart of extracting patients information in our study.

**Supplementary Figure 2:** Association of LNM with age of patients with IPMN was identified by measuring the p value of linear trend.

**Supplementary Figure 3:** Comparison of CSS and OS among patients diagnosed in 2004-2009 and 2010-2015 years. OS (**A**) and CSS (**B**).

**Supplementary Figure 4:** OS (**A**) and CSS (**B**) of patients aged 20–39 years, 40–49 years, 50–59 years, 60–69 years, 70-79 years and 80+ years were compared by K-M curves.

**Supplementary Figure 5:** The Association between our extrected clinical characteristics of patients and survival was analysed by Lasso regression analysis.
